# QuickStats

**Published:** 2013-05-10

**Authors:** Linda F. McCaig, Esther Hing

**Figure:**
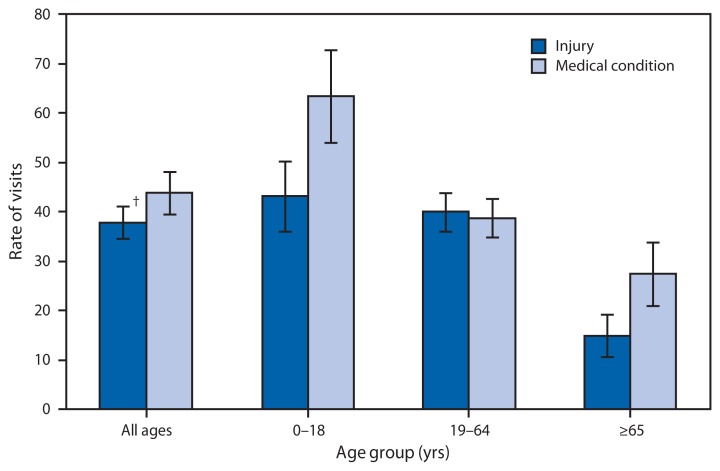
Average Annual Rate of Eye-Related Emergency Department Visits for Injuries and Medical Conditions,* by Age Group — United States, 2007–2010 * Per 10,000 population, based on 4-year annual average. ^†^ 95% confidence interval.

During 2007–2010, an average of 2.4 million eye-related visits were made to emergency departments (EDs) each year. During this period, 43.7 visits per 10,000 persons were the result of medical conditions, and 37.6 visits per 10,000 persons were the result of injuries. Significant differences in the reason for eye-related ED visits were observed by age group. Children and persons aged ≥65 years were more likely to visit the ED for an eye-related medical condition than an eye injury. The eye-related visit rate for a medical condition was highest among those aged ≤18 years (63.3 per 10,000 persons) and lowest among those aged ≥65 years (27.3).

**Source:** National Hospital Ambulatory Medical Care Survey. Available at http://www.cdc.gov/nchs/ahcd.htm.

